# One cycle of nelarabine given after induction is well tolerated but does not improve outcome in adults with T‐cell precursor acute lymphoblastic leukemia: Outcome data from the UKALL14 randomized‐controlled trial

**DOI:** 10.1002/hem3.70480

**Published:** 2026-10-01

**Authors:** Clare J. Rowntree, Amy A. Kirkwood, Nadine Farah, Emma Lawrie, David I. Marks, Andrew K. McMillan, Tobias F. Menne, Anthony V. Moorman, Bela Patel, Pip Patrick, Paul Smith, Krisztina Z. Alapi, Lingyi Wang, Marc R. Mansour, Adele K. Fielding

**Affiliations:** ^1^ Cardiff and Vale UHB Cardiff United Kingdom; ^2^ CRUK and UCL Cancer Trial Centre, Cancer Institute UCL London United Kingdom; ^3^ UCL Cancer Institute London United Kingdom; ^4^ United Bristol Healthcare Trust Bristol United Kingdom; ^5^ Centre for Clinical Haematology Nottingham City Hospital Nottingham United Kingdom; ^6^ Newcastle Upon Tyne Hospitals NHS Foundation Trust Newcastle upon Tyne United Kingdom; ^7^ Research Cytogenomics Group, Translational and Clinical Research Institute Newcastle University Newcastle upon Tyne United Kingdom; ^8^ Barts Cancer Institute London United Kingdom; ^9^ Queen Mary University of London, Specialist Integrated Haematological Malignancy Diagnostic Service London United Kingdom; ^10^ Developmental Biology and Cancer UCL Great Ormond Street Institute of Child Health London United Kingdom; ^11^ Centre for Blood Research, Hull York Medical School University of York York United Kingdom

## Abstract

UKALL14 (NCT01085617) T‐cell arm was a phase 3, randomized‐controlled trial. Adults aged 25–65 years were randomized 1:1 at entry to receive standard of care (SOC) or SOC + nelarabine 1.5 g/m^2^ (Days 1, 3, and 5) to be administered following second induction. The primary end‐point was event‐free survival (EFS). From 2011 to 2018, 143 patients were randomly assigned to SOC (*N* = 75) or SOC + nelarabine (*N* = 68). There was no significant difference in EFS between the treatment groups; HR 0.86 (95% CI 0.52–1.43) P = 0.57, EFS: 58.2% SOC versus 62.7% SOC + nelarabine. The severe adverse events and nonrelapse mortality rates did not differ between the arms; in particular, there was no difference in grade 3–4 neurotoxicity. The 3‐year cumulative incidence of relapse was 31% in the SOC arm versus 28.9% in the SOC+ nelarabine arm. Forty of 44 relapses (90.9%) occurred within 2 years of randomization; the median survival postrelapse was 5.7 months, with no difference by arm. Diagnostic samples were analyzed for level of differentiation arrest by analysis of the TCRg locus to detect the most immature cases through absence of biallelic TCRg deletion (ABD). There was a trend toward more relapses with ABD (37.3%) versus no ABD (19.5%), P = 0.066. Diagnostic samples (*N* = 133) were analyzed for mutations in *NOTCH1, FBXW7, NRAS, KRAS,* and *PTEN*, with no significant differences in CR rates or survival in patients with wild‐type (WT) *NOTCH1* or *FBXW7*. The 12 patients with *NRAS* mutations had significantly inferior CR and EFS compared to patients with wild‐type *NRAS*.

## INTRODUCTION

T‐cell acute lymphoblastic leukemia (T‐ALL) is an uncommon disorder, comprising approximately 25% of ALL in adults. Although outcomes for adults with T‐ALL have improved, relapsed/refractory disease is seen in over 30% of patients treated on modern protocols,^1^, ^2^ and outcomes for these patients remain poor.^3^ Nelarabine is the only new drug that has been licensed for use in T‐ALL in the past 20 years. Initial pediatric trials reported response rates of 14%–55% to single‐agent nelarabine when used in patients with relapsed/refractory (rel/ref) T‐ALL and lymphoblastic lymphoma.^4^, ^5^ Phase 2 trials in adults with rel/ref T‐ALL reported similarly encouraging response rates (41%–46%) to single‐agent nelarabine, with 28% 1‐year overall survival (OS).^6^, ^7^ Use of nelarabine in combination with other chemotherapy agents also resulted in promising response rates in children with rel/ref disease.^8^, ^9^ A concerning side effect reported in these trials was neurotoxicity, affecting about 1 in 5 patients, although grade 3–4 toxicities were only observed in 7% and were mostly transient and reversible.

Differentiating between patients with T‐ALL who are likely to achieve a good outcome with chemotherapy‐only approaches and those in whom allogeneic stem cell transplantation (alloSCT) is required is challenging. Risk assessment has traditionally relied on presenting factors such as white cell count (WCC) together with minimal residual disease (MRD) responses after induction therapy.^10^, ^11^ Early T‐cell precursor (ETP) T‐ALL, as defined by immunophenotype, has been previously reported to confer a worse outcome in both pediatric and adult patients.^12^, ^13^ However, immunophenotyping underestimates the number of patients with an ETP gene expression signature.^14^, ^15^, ^16^ An alternative molecular analysis based on the V–J recombination status of the T‐cell receptor (TRG) gene has been described^14^ that overlaps with ETP status. The majority of patients with absence of biallelic deletion (ABD) of the TRG locus have an ETP‐ALL gene expression signature.^15^ ABD has been shown to predict early treatment failure in pediatric T‐ALL,^14^ although others have not found it to be a poor prognostic factor when patients are treated on an MRD risk‐adapted protocol.^17^

In 2013, the GRAALL study group published a 5‐gene risk classifier for T‐ALL, demonstrating that mutations in *NOTCH1* or *FBXW7* in the absence of *N/K‐RAS* or *PTEN* mutations yielded superior outcomes compared to those with wild‐type *NOTCH1*/*FBXW7* with or without mutations in *N/K RAS/PTEN*.^2^, ^18^ This classifier does not have prognostic impact in all patient cohorts, potentially due to differences in treatment regimens.^19^, ^20^, ^21^ However, mutations in *N/K RAS* have consistently been shown to be predictive of poor outcomes in adults with T‐ALL.^22^

Here, we report the final analysis of the UKALL14 trial for adults with T‐ALL aged 25‐65 years who were randomized at diagnosis to receive one cycle of nelarabine to be administered after induction phase 2 of the standard of care (SOC) therapy. We also assessed the prognostic impact of ABD status and the GRAALL 5‐gene oncogenic classifier within the whole patient cohort and in relation to randomized therapy.

## METHODS

### Patients and treatment

UKALL14 (NCT01085617) was a UK‐wide, investigator‐initiated, phase 3, randomized‐controlled trial with two randomizations as well as two single‐arm questions on the use of pegylated asparaginase and the role of reduced‐intensity conditioned (RIC) alloSCT in adults aged 41 years and older. The outcomes of the B‐cell randomization and RIC alloSCT question have been previously published.^23^, ^24^

Eligible patients were aged 25–65 years with *de novo* T‐ALL (>25% lymphoblasts in the bone marrow). There were minimal exclusions from trial entry: HIV infection, active hepatitis B infection, pregnancy or lactation, and transformation of chronic myeloid leukemia. Assessment of a patient's fitness to receive intensive treatment with curative intent was left up to the treating clinicians. Although patients were randomized at diagnosis, nelarabine was administered after recovery from 2 cycles of induction therapy, and patients were not eligible to receive nelarabine if they experienced neurotoxicity grade 2 or greater due to any cause postinduction. Patients with detectable CNS disease at diagnosis were eligible for randomization.

There were no allowable dose adjustments to nelarabine, but adjustments to standard‐of‐care medications were permitted for organ dysfunction or laboratory test abnormalities, according to the summary of product characteristics.

The trial was managed by The Cancer Research UK and University College London Trials Centre (UCL CTC), and all UK National Health Service centers treating patients with ALL participated (65 centers in total; Supporting Information S1: Table S1). Ethical approval was obtained from the London‐Fulham Research and Ethics Committee. Written informed consent was obtained in accordance with the Declaration of Helsinki.

Patients were randomly assigned (1:1) using a computer‐based system at diagnosis to SOC or SOC plus nelarabine (SOC + N) at UCL CTC using minimization and stratified by sex, age (≤40 vs >40 years), and WCC (<100 × 10^9^ cells per L vs. ≥100 × 10^9^ cells per L). No masking was used for patients, treating clinicians, or UCL CTC staff, including the trial statistician. The staff analyzing MRD, ABD status, and 5‐gene classifier were masked to treatment allocation.

The UKALL14 SOC treatment has been previously published,^23^ but is shown schematically for the T‐cell arm in Supporting Information S1: Figure 1. In brief, all patients were treated with two cycles of induction chemotherapy. Ig/TCR MRD was quantified by a single central lab according to EuroMRD criteria, on recovery of peripheral blood counts after the second course of induction therapy. A risk assessment determined whether patients should proceed to alloSCT (myeloablative for patients aged 40 years and under, and nonmyeloablative for patients over 41 years; the conditioning regimen was specified by protocol, Supporting Information S1: Figure 1) or should continue with intensification, consolidation, and maintenance chemotherapy. High risk was defined as age >40, presenting WCC ≥100, high‐risk cytogenetics (*KMT2A::AFF1*, low hypodiploidy/near triploidy [<40 chromosomes] or complex karyotype [defined by more than 5 abnormalities]), or MRD >1 × 10^−4^. Patients with standard‐risk ALL and a fully matched sibling donor were permitted to receive alloSCT, per patient and clinician choice. Nelarabine 1.5 g/m^2^ was administered on Days 1, 3, and 5 following peripheral blood count recovery post‐induction phase 2. The trial protocol and statistical analysis plan can be accessed via the UCL Cancer Trial Centre.

### Sample processing, sequencing, and analysis

All patient samples were processed by the UK Adult ALL MRD laboratory at the UCL Cancer Institute. DNA was extracted from mononuclear cells obtained from diagnostic bone marrow or peripheral blood samples as previously described^25^ and was sent for whole‐exome sequencing at the Wellcome Sanger Institute using the SureSelectXT Human All Exon V6 + UTR (5190‐8881) capture panel by Agilent (Agilent Technologies LDA UK Limited). To determine ABD status, deletions of the *TRG* g locus were quantified by quantitative PCR using primers within the intron between the V and J regions of the *TRG* locus as compared to the reference gene *ANLN* as described previously.^17^ The whole‐exome paired‐end DNA sequencing reads were preprocessed using nf‐core/sarek (v2.6.1 and v3.1.2).^26^ This included alignment of raw FASTQ files to the human reference genome (GRCh38) using the built‐in BWA‐MEM aligner,^27^ followed by duplicate marking and base recalibration according to the GATK best practices. Read coverage was evaluated using DepthOfCoverage^28^ (GATK v4.2.5.0). Variant calling was performed using Sarek (v3.1.2)^26^ with Mutect2 in tumor‐only mode. A subset of post‐induction remission samples (*N* = 50) with MRD <5% was used to construct a panel of nonleukemic controls to filter out sequencing artifacts during variant calling. The resulting VCF files were annotated using Funcotator^28^ (GATK v4.2.5.0) and subsequently filtered using MAFTOOLS (v2.14.0)^29^ based on the following criteria: (1) ClinVar significance; (2) tumor allele frequency ≥5%; (3) tumor allele read count ≥5; and (4) minor allele frequency <0.001 in gnomAD (af‐only‐gnomad.hg38.vcf.gz) and dbSNP (dbsnp_146.hg38.vcf.gz). The resulting driver mutations were further verified by pairwise variant calling using Mutect2 (GATK v4.2.5.0) on 50 patients with matched PP2 samples (MRD <5%), and all were manually reviewed in IGV (v2.12.3).^30^

### Statistics

EFS was assumed to be around 50% at 3 years in the SOC arm and we aimed to show an improvement to 75% with the addition of Nelarabine (i.e., an HR of 0.42). Forty‐five events would yield 86% power to show this difference with a 2‐sided alpha of 0.05. 144 patients recruited over 6 years with at least 6 months of follow‐up after the last randomization would allow us to see these events.

The primary endpoint was event‐free survival (EFS). The secondary endpoints were complete remission (CR) rate at the end of induction phase 2, OS, cumulative incidence of relapse (CIR) including bone marrow and CNS relapses, nonrelapse mortality (NRM), and MRD at the end of induction phases 1 and 2.

EFS, OS, and NRM times were measured from the date of random assignment until the date of event; relapse (>5% blasts in the bone marrow, T lymphoblasts in cerebral spinal fluid or biopsy of extramedullary tissue, local assessment); or death for EFS and NRM or death for OS. Patients without an event were censored at the date last seen. Time to relapse was measured from the date of remission until the date of relapse, with patients not experiencing relapse censored at the date last seen or death. EFS and OS were analyzed using Kaplan–Meier survival analysis with groups compared using Cox regression and the log‐rank test. Time to relapse and NRM were analyzed using competing risks survival analysis using the method of Fine and Grey, with NRM and relapse treated as competing risks, respectively. Site‐specific relapse also included relapse at other sites as competing events. All discrete data (remission, MRD, and adverse events) were compared using Fisher's exact tests and continuous data were compared using Wilcoxon–Mann–Whitney tests. The primary analysis population is the intention‐to‐treat population recruited after a 2012 protocol amendment, which changed the chemotherapy backbone in induction^31^ with a per‐protocol analysis also performed for patients receiving their randomized allocation. The main outcome analyses for whole cohort are included in Supporting Information S1: Table S2. All patients were included in the analyses of ABD status and the 5‐gene classifier. All analyses were performed using STATA version 19.5 (STATAcorp.).

## RESULTS

Between February 2011 and July 2018, 156 patients with *de novo* T‐ALL were recruited from 51 of the 65 participating UK centers and were randomized to receive either SOC (*N* = 81) or SOC + N (*N* = 75). The primary analysis includes *N* = 143 (*N* = 75 SOC and *N* = 68 SOC + N) recruited after the April 2012 protocol amendment.^31^ Table 1 shows the patient characteristics, which were generally balanced for high‐risk factors, including age and presenting white cell count, although there were more cytogenetic high‐risk factors seen in SOC (*N* = 12, 19.4%) vs. SOC + N (*N* = 4, 7.7%). One hundred and eight (75.5%) patients had extramedullary disease (including lymphadenopathy) at diagnosis, with 4 (2.8%) having CNS involvement.

**Table 1:** Patient characteristics.

	SOC	SOC + N	Total
	*N* = 75	*N* = 68	*N* = 143
Age (years), median (IQR)	38 (29−51)	36 (28−47)	38.0 (29−51)
Range	25−61	25−64	25−61
Age group, *N* (%)			
40 or under at randomization	41 (54.7)	38 (55.9)	79 (55.2)
Over 40 at randomization	34 (45.3)	30 (44.1)	64 (44.8)
Sex, *N* (%)			
Male	53 (70.7)	38 (55.9)	79 (55.2)
Female	22 (29.3)	30 (44.1)	64 (44.8)
ECOG, *N* (%)			
0	50 (66.7)	42 (62.7)	92 (64.8)
1	21 (28.0)	20 (29.9)	41 (28.9)
2	2 (2.7)	4 (6.0)	6 (4.2)
3	2 (2.7)	1 (1.5)	3 (2.1)
Missing	0	1	1
Baseline WBC group, *N* (%)			
<30	41 (54.7)	33 (48.5)	74 (51.7)
30–100	17 (22.7)	19 (27.9)	36 (25.2)
≥100	17 (22.7)	16 (23.5)	33 (23.1)
Median (IQR)	16.5 (7.5−76.2)	33.0 (10.75−95.55)	16.5 (7.5−76.2)
Range	0.36−427.05	0.5−380.3	0.36−427.05
Confirmed CNS disease, *N* (%)	1 (1.3)	3 (4.4)	4 (2.8)
Other extramedullary involvement, *N* (%)	54 (72.0)	54 (79.4)	108 (75.5)
t(4;11) (q21;q23), *N* (%)			
Absent	70 (100.0)	53 (100.0)	123 (100.0)
Missing	5	15	20
t(11q23), *N* (%)			
Absent	65 (98.5)	52 (100.0)	117 (99.2)
Present	1 (1.5)	0	1 (0.8)
Missing	9	16	25
HOTR or near haploidy, *N* (%)			
Absent	55 (100.0)	46 (95.8)	101 (98.1)
Present	0	2 (4.2)	2 (1.9)
Missing	20	20	40
Complex karyotype, *N* (%)			
Absent	46 (80.7)	45 (95.7)	91 (87.5)
Present	11 (19.3)	2 (4.3)	13 (12.5)
Missing	18	21	39
Any UKALL 14 cytogenetic risk factor, *N* (%)			
Absent	44 (80.0)	43 (91.5)	87 (85.3)
Present	11 (20.0)	4 (8.5)	15 (14.7)
Missing	20	21	41
Cytogenetic group, *N* (%)			
Complex karyotype	11 (17.7)	2 (3.8)	13 (11.4)
High hyperdiploid	1 (1.6)	0	1 (0.9)
KMT2A other	1 (1.6)	0	1 (0.9)
Low hypodiploid/near‐triploid	0	2 (3.8)	2 (1.8)
Other	49 (79.0)	48 (92.3)	97 (85.1)
Missing	13	16	29
Cytogenetic risk group, *N* (%)			
Genetic good risk	1 (1.6)	0	1 (0.9)
Standard risk	49 (79.0)	48 (92.3)	97 (85.1)
High risk	12 (19.4)	4 (7.7)	16 (14.0)
Missing	13	16	29
Baseline UKALL 14 risk, *N* (%)			
Standard risk	19 (25.3)	20 (29.4)	39 (27.3)
High risk^a^	48 (64.0)	42 (61.8)	90 (62.9)
Assumed standard—missing cytogenetic data	8 (10.7)	6 (8.8)	14 (9.8)
Baseline UKALL 14 risk (no age), *N* (%)			
Standard risk	35 (46.7)	31 (45.6)	66 (46.2)
High risk^a^	26 (34.7)	18 (26.5)	44 (30.8)
Assumed Standard ‐ missing cytogenetic data	14 (18.7)	19 (27.9)	33 (23.1)

a Age ≥41, WCC ≥100, or high‐risk cytogenetics; KMT2A/AFF1, low hypodiploidy/near triploidy (HoTr, <40 chromosomes), and complex cytogenetics (>5 abnormalities).

The consort diagram is shown in Figure 1. Of the 68 eligible patients randomized at diagnosis to receive SOC+N, only 47 (69.1%) received nelarabine. Six patients exited the trial prior to induction phase 2 (one death, two due to lack of response, two due to toxicity, and one due to withdrawal of consent) and a further 15 patients did not receive nelarabine due to neurotoxicity at the time at which treatment was due (*N* = 8), patient choice (*N* = 3), hematological toxicity (*N* = 2), clinician choice (*N* = 1), or death in induction 2 (*N* = 1). Forty‐two patients (61.8%) received all three planned doses, with five patients stopping after two doses, two due to neurotoxicity, one due to suspected neurotoxicity (ultimately reported as myositis), one due to hepatotoxicity, and one because the drug was not available. Of the two patients who stopped nelarabine early due to neurotoxicity, one patient had grade 2 leukoencephalopathy and the other had grade 1 peripheral neuropathy. AlloSCT was received by 88 patients, 54 in the SOC arm (72%) and 34 in the SOC + N arm (50%), of whom 69 were administered the per trial‐specified conditioning regimen (42 SOC and 27 SOC + N), with 19 receiving a nonspecified alloSCT conditioning protocol (12 SOC and 7 SOC + N).

**Figure 1:**
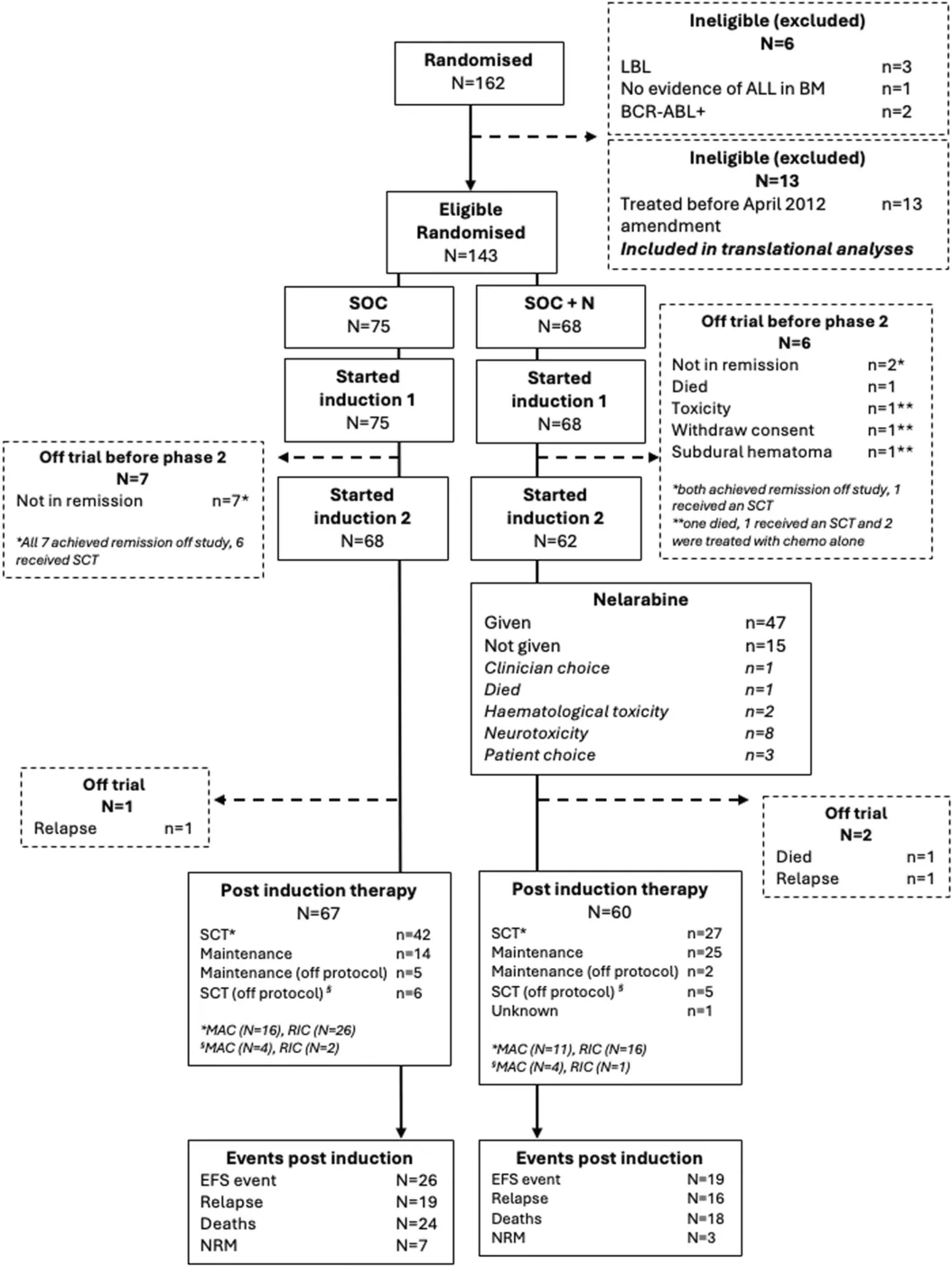
Consort diagram.

Outcomes are shown in Table 2. The end of induction 2 CR rate was 94.1% in the SOC + N arm and 90.7% in the SOC arm (P = 0.54). There was no significant difference in the numbers of patients in molecular remission (negative or POQR) by Ig/TCR quantification (*N* = 42.7% in SOC + N vs. 44% SOC P > 0.99) at the end of induction 2. At a median follow‐up of 80.3 months (IQR: 63.1–99.9), 44 of the 143 patients treated on trial had relapsed and 16 patients had died without relapse reported (including 1 phase 1 induction death), providing 60 EFS events; the median EFS had not been reached in either arm. There was no significant difference in EFS between the treatment groups, HR 0.86 (95% CI: 0.52–1.43), P = 0.57 with 3‐year EFS rates of 62.7% (50.0–73.0) SOC + N compared to 58.2% with SOC (95% CI 46.1%–68.4%) (Figure 2), and the preplanned subgroup analysis did not show any differential response between the arms, as shown in the Forest plot in Figure 3. OS was also similar between arms, HR 0.90 (0.53–1.51), P = 0.68, with 3‐year rates of 68.7% (56.1–78.3) vs. 63.6% (51.5–73.8), respectively (Figure 2). A per‐protocol analysis included all patients who received at least one dose of Nelarabine in the SOC + N arm and SOC arm patients who were alive, in remission, and without persistent neurotoxicity (grade ≥2); the difference remained nonsignificant: HR 0.83 (0.44–1.,55), P = 0.55, and 3‐year EFS rates 65.1% (49.5–77.0) versus 61.9% (48.8–72.6).

**Table 2:** Patient outcomes.

	SOC	SOC + N
	*N* = 75	*N* = 68
In remission by the end of phase 1, *N* (%)	60 (81.1)	59 (86.8)
In remission by the end of phase 2, *N* (%)^a^	68 (90.7)	64 (94.1)
	P = 0.54	
MRD‐negative remission by the end of phase 1, *N* (%)^b^	16 (21.3)	17 (25.0)
Immunoglobulin heavy chain/T‐cell receptor	3.8 × 10^−4^ (1 × 10^−6^–7.9 × 10^−3^)	2.6 × 10^−4^ (5.5 × 10^−6^–2.0 × 10^−3^)
MRD quantification (phase 1)^c^	1 × 10^−6^–1.00	1 × 10^−6^–0.52
MRD‐negative remission in induction, *N* (%)^a^^,^ b	33 (44.0)	29 (42.7)
	P > 0.99	
Immunoglobulin heavy chain/T‐cell receptor	1 × 10^−6^ (1 × 10^−6^−1 × 10^−5^)	1 × 10^−6^ (1 × 10^−6^−1 × 10^−5^)
MRD quantification (phase 2)	1 × 10^−6^ 0.061	1 × 10^−^^6^–1.4 × 10^−3^
	P = 0.70	
Patients at high risk by MRD alone	3 (4%)	1 (1.5%)
Event‐free survival		
Number of events/patients	33/75	27/68
HR (95% CI)	0.86 (0.52–1.43)	
P‐value	P = 0.57	
3‐year EFS rate (95% CI)	58.2% (46.1–68.4)	62.7% (50.0–73.0)
Adjusted for baseline risk factors^d^		
HR (95% CI)	0.92 (0.49–1.69)	
P‐value	P = 0.78	
Baseline risk factors^d^ and SCT as time‐varying		
HR (95% CI)	0.86 (0.46–1.61)	
P‐value	P = 0.64	
Per‐protocol		
Number of events/patients	25/63	16/47
HR (95% CI)	0.83 (0.44–1.55)	
P‐value	P = 0.55	
3‐year EFS rate (95% CI)	61.9% (48.8–72.6)	65.1% (49.5–77.0)
Adjusted for baseline risk factors^d^		
HR (95% CI)	0.88 (0.41–1.88)	
P‐value	P = 0.74	
Baseline risk factors^d^ and SCT as time‐varying		
HR (95% CI)	0.81 (0.37–1.76)	
P‐value	P = 0.59	
Overall survival		
Number of events/patients	31/75	26/68
HR (95% CI)	0.90 (0.53–1.51)	
P‐value	P = 0.68	
3‐year OS rate (95%CI)	63.6% (51.5–73.4)	68.7% (56.1–78.3)
Relapse^e^		
Number of events/patients	25/75	19/67
HR (95% CI)	0.81 (0.45–1.48)	
P‐value	P = 0.50	
3‐year relapse rate (95% CI)	31.0% (21.8–42.9)	28.9% (19.5–41.5)
Sites of relapse		
Isolated bone marrow	15	9
Isolated CNS	2	7
Bone marrow and other site^f^	3	2
Extramedullary alone^g^	4	1
Unknown	1	0
Bone marrow relapse^e^		
Number of events/patients	18/75	11/67
HR (95% CI)	0.62 (0.29–1.32)	
P‐value	P = 0.21	
3‐year BM relapse rate (95% CI)	23.3% (15.2–34.8)	16.8% (9.7–28.3)
CNS relapse^e^		
Number of events/patients	3/75	7/67
HR (95% CI)	2.66 (0.69−10.26)	
P‐value	P = 0.14	
3‐year CNS relapse rate (95% CI)	4.1% (1.3–12.1)	10.6% (5.2–20.9)
Nonrelapse mortality^h^		
Number of events/patients	8/75	7/67
HR (95% CI)	0.98 (0.35–2.70)	
P‐value	P = 0.97	
3‐year NRM rate (95% CI)	10.8% (5.5–20.4)	7.5% (3.2–17.2)
Second malignancies^i^		
Number of events/patients	6/75	4/68
HR (95% CI)	0.75 (0.21–2.66)	
P‐value	P = 0.66	
3‐year SM rate (95% CI)	4.1% (1.3–12.1)	1.5% (0.2–10.1)

^a^ 30/47 of the patients administered Nelarabine underwent response assessment after Nelarabine was administered; 3 had not achieved remission in phase 1, that is, could have gone into remission due to Nelarabine, with 6 going from MRD‐positive to ‐negative/POQR.

^b^ Patients who are both in remission and MRD‐negative or POQR (positive outside of the quantitative range). The denominator includes all patients, including those who are missing response or MRD data (*N* = 1 for response, *N* = 56 for MRD in phase 1, and 72 in phase 2).

^c^ MRD negative values set as 1 × 10^−6^, POQR as 1 × 10^−5^, and value >1 set as 0.99999.

^d^ Age, sex, WCC, genetic risk group, extramedullary involvement, and BMI.

^e^ Analyses by site, censor one patient with an unknown site of relapse at the date last known to be in remission.

^f^ SOC arm Bone marrow, CNS, Testes, and unspecified other site (*N* = 1), bone marrow and mediastinum (*N* = 1), bone marrow and breast (*N* = 1), SOC + N: bone marrow and neck, mediastinum, and liver (*N* = 1), and bone marrow and mediastinal (*n* = 1).

^g^ SOC: right neck, lung, mediastinum pleura, and Gerota's fascia bilaterally (*N* = 1), skin punch biopsy left iliac fossa (*N* = 1), infiltration of ocular muscles + myocardium (*N* = 1), and neck lymph nodes (*N* = 1). SOC + N: mediastinal mass (*N* = 1).

^h^ This excludes one patient who never achieved remission.

^i^ This excludes nonmelanoma skin cancer (*N* = 1). Events were AML (*N* = 2 SOC + N), Head and neck (*N* = 1, SOC + N), Melanoma (*N* = 1, SOC), MDS (*N* = 1 SOC, *N* = 1 SOC + N), PTLD (*N* = 2, SOC), Prostate (*N* = 1 SOC), and Renal (*N* = 1 SOC), and all occurred before relapse.

**Figure 2:**
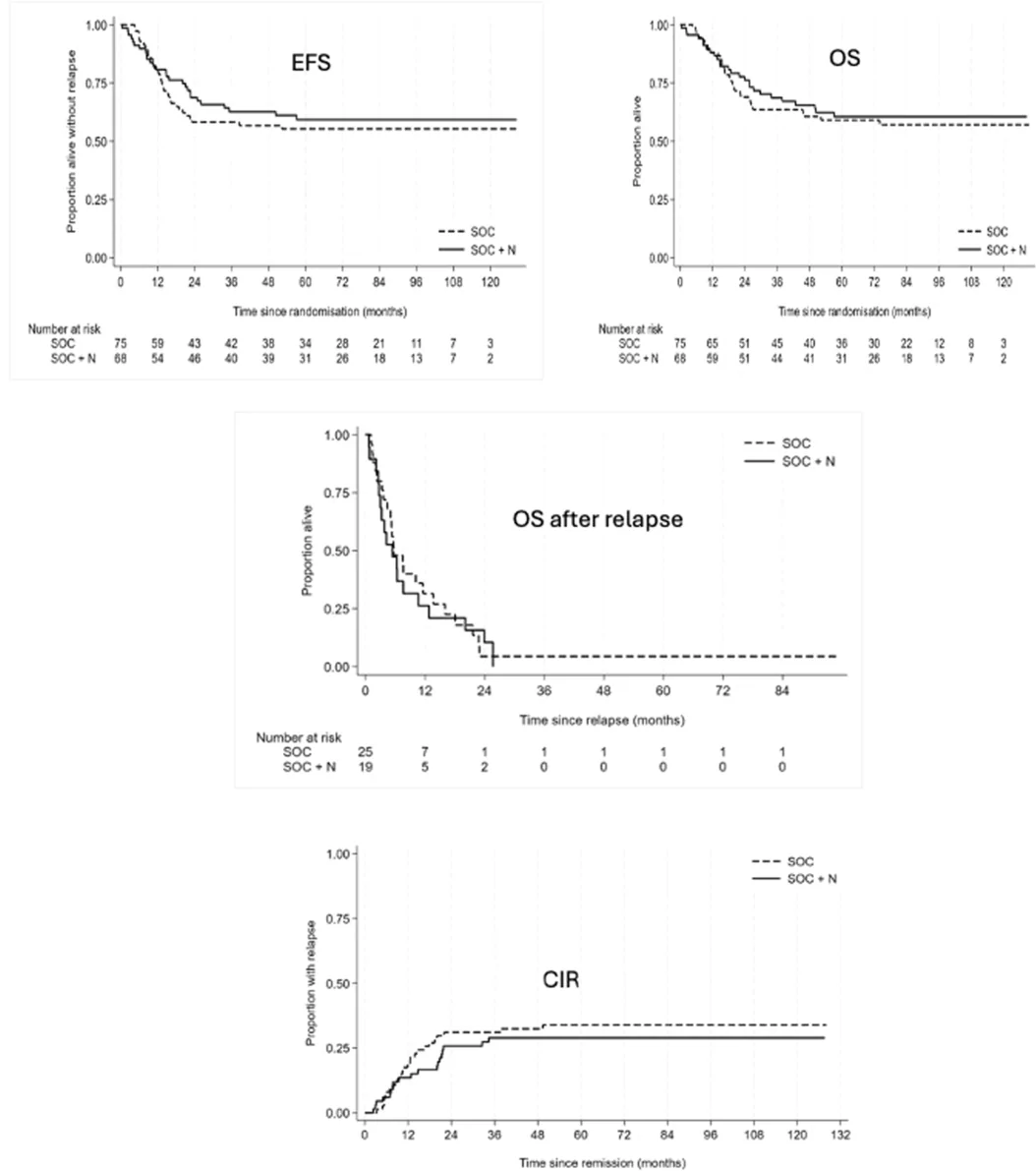
**(A)** Kaplan–Meier event‐free survival by randomized arm. **(B)** Kaplan–Meier overall survival by randomized arm. **(C)** Kaplan–Meier overall survival postrelapse by randomized arm. **(D)** Kaplan–Meier cumulative incidence of relapse by randomized arm.

**Figure 3:**
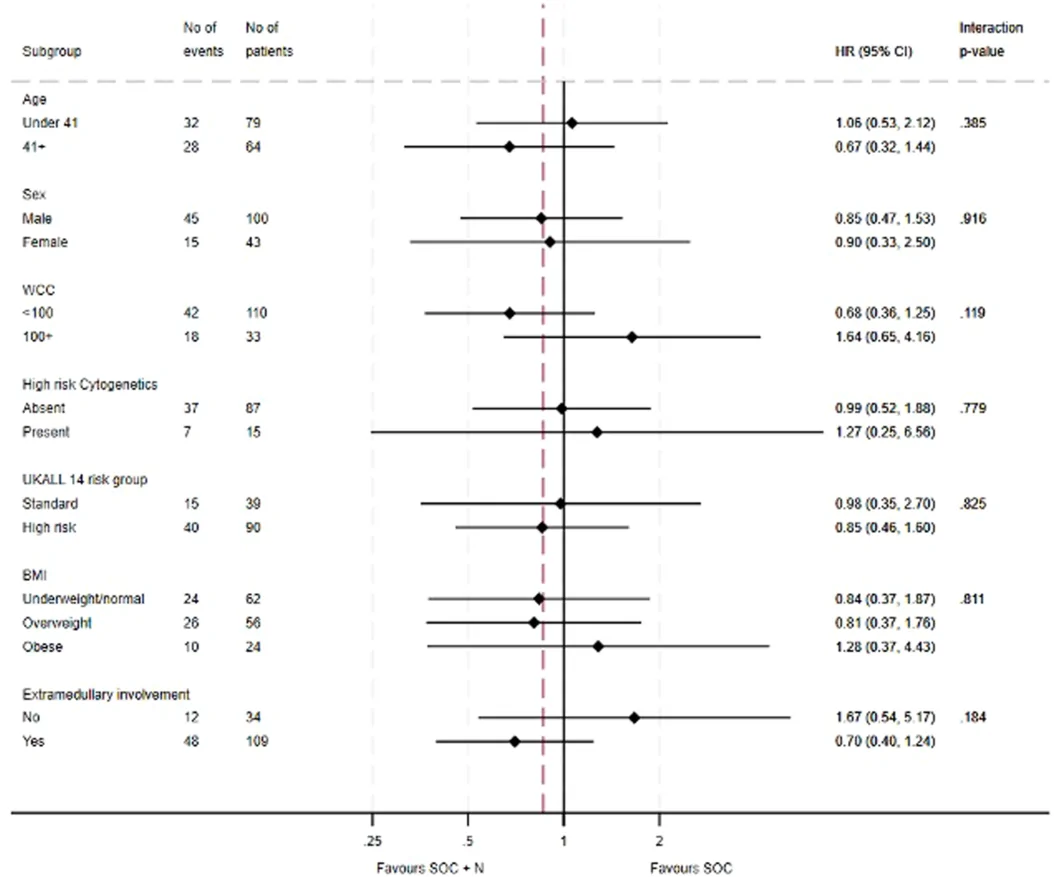
Forest plot.

The rate of severe adverse events and nonrelapse mortality did not differ between the arms, in any phase of therapy administered, nor was there any difference in grade 3–4 neurotoxicity (6 cases in the SOC arm and 7 cases in the SOC + N arm) in induction phase 2. No deaths were reported from neurotoxicity in either arm. Fifteen patients were reported to have died in remission, with no significant difference by arm: HR 0.98 (95% CI: 0.35–0.70) and P = 0.97. The cumulative incidence of NRM at 3 years was 7.5% (3.2–17.2) in the SOC +N arm versus 10.8% (5.5–20.4) in the SOC arm. Table 3 summarizes the grade 3–4 adverse events (AEs) reported by phase of treatment (grades 3–5 for induction phase 2). There was no difference between SOC + N and SOC.

**Table 3:** Summary of toxicity by randomized arm.

	Standard phases 1 & 2	Standard phases 1 & 2 + N
	*N* = 75	*N* = 68
Phase 1 induction		
Any grade 3–4 event	67 (91.8%)	59 (90.8%)
Grade 3–4 infection	13 (17.8%)	7 (10.8%)
Grade 3–4 nervous system disorders	8 (11.0%)	5 (7.7%)
On‐therapy mortality	0	2
Sepsis	0	2
Patients who stopped trial treatment due to toxicity	0	2
Phase 2 induction		
Any grade 3–4 event	64 (95.5%)	55 (93.2%)
	P = 0.71	
Grade 3–4 infection	27 (40.3%)	16 (27.1%)
	P = 0.14	
Grade 3–4 nervous system disorders	6 (9.0%)	7 (11.9%)
	P = 0.77	
On‐therapy mortality 2		
Multi‐organ failure	0	1
Patients who stopped trial treatment due to toxicity	1	2
Patients stopped treatment due to death	0	1

All grade 3–5 events in phase 2 induction	*N* = 67		*N* = 59
System organ class\adverse event	Grade 3–4	Grade 5	Grade 3–4	Grade 5
Blood and Lymphatic System Disorders	46 (68.7)	0	41 (69.5)	0
Anemia	43 (64.2)	0	35 (59.3)	0
Bone marrow hypocellular	0	0	1 (1.7)	0
Febrile neutropenia	21 (31.3)	0	21 (35.6)	0
Cardiac disorders	3 (4.5)	0	0	0
Pericardial effusion	1 (1.5)	0	0	0
Sinus tachycardia	2 (3.0)	0	0	0
Supraventricular tachycardia	1 (1.5)	0	0	0
Eye disorders	0	0	1 (1.7)	0
Optic nerve disorder	0	0	1 (1.7)	0
Gastrointestinal disorders	8 (11.9)	0	4 (6.8)	0
Ascites	1 (1.5)	0	0	0
Diarrhea	0	0	1 (1.7)	0
Nausea	4 (6.0)	0	2 (3.4)	0
Typhlitis	1 (1.5)	0	0	0
Vomiting	1 (1.5)	0	2 (3.4)	0
Other: Parotiditis	1 (1.5)	0	0	0
General disorders and administration site conditions	7 (10.4)	0	4 (6.8)	1 (1.7)
Fever	6 (9.0)	0	3 (5.1)	0
Infusion‐related reaction	1 (1.5)	0	0	0
Multi‐organ failure	0	0	0	1 (1.7)
Pain	0	0	2 (3.4)	0
Infections and infestations	27 (40.3)	0	16 (27.1)	0
Anorectal infection	0	0	1 (1.7)	0
Appendicitis	1 (1.5)	0	1 (1.7)	0
Catheter‐related infection	1 (1.5)	0	1 (1.7)	0
Device‐related infection	4 (6.0)	0	6 (10.2)	0
Lung infection	2 (3.0)	0	2 (3.4)	0
Sepsis	5 (7.5)	0	6 (10.2)	0
Skin infection	2 (3.0)	0	0	0
Small intestine infection	1 (1.5)	0	0	0
Tooth infection	3 (4.5)	0	0	0
Upper respiratory infection	2 (3.0)	0	1 (1.7)	0
Other: Bacterial infection NOS	3 (4.5)	0	2 (3.4)	0
Other: Meningitis	0	0	1 (1.7)	0
Other: Septic arthritis	1 (1.5)	0	0	0
Other: Unknown	5 (7.5)	0	0	0
Other: Viral infection NOS	0	0	1 (1.7)	0
Investigations	60 (89.6)	0	50 (84.7)	0
Activated partial thromboplastin time prolonged	0	0	1 (1.7)	0
Alanine aminotransferase increased	8 (11.9)	0	4 (6.8)	0
Alkaline phosphatase increased	1 (1.5)	0	3 (5.1)	0
Aspartate aminotransferase increased	0	0	1 (1.7)	0
Blood bilirubin increased	1 (1.5)	0	3 (5.1)	0
GGT increased	1 (1.5)	0	0	0
Granulocytopenia	8 (11.9)	0	4 (6.8)	0
Lymphocyte count decreased	3 (4.5)	0	2 (3.4)	0
Myelosuppression	8 (11.9)	0	4 (6.8)	0
Neutrophil count decreased	57 (85.1)	0	45 (76.3)	0
Pancytopenia	11 (16.4)	0	4 (6.8)	0
Platelet count decreased	10 (14.9)	0	11 (18.6)	0
Weight loss	0	0	2 (3.4)	0
White blood cell decreased	50 (74.6)	0	39 (66.1)	0
Metabolism and nutrition disorders	7 (10.4)	0	5 (8.5)	0
Anorexia	2 (3.0)	0	2 (3.4)	0
Hypertriglyceridemia	1 (1.5)	0	0	0
Hypoalbuminemia	2 (3.0)	0	1 (1.7)	0
Hypocalcemia	0	0	1 (1.7)	0
Hypokalemia	2 (3.0)	0	1 (1.7)	0
Musculoskeletal and connective tissue disorders	0	0	1 (1.7)	0
Myositis	0	0	1 (1.7)	0
Nervous system disorders	6 (9.0)	0	7 (11.9)	0
Dizziness	0	0	1 (1.7)	0
Headache	4 (6.0)	0	5 (8.5)	0
Peripheral motor neuropathy	1 (1.5)	0	1 (1.7)	0
Peripheral sensory neuropathy	0	0	1 (1.7)	0
Seizure	0	0	1 (1.7)	0
Syncope	1 (1.5)	0	0	0
Psychiatric disorders	3 (4.5)	0	2 (3.4)	0
Agitation	1 (1.5)	0	1 (1.7)	0
Anxiety	1 (1.5)	0	0	0
Depression	2 (3.0)	0	0	0
Insomnia	0	0	1 (1.7)	0
Reproductive system and breast disorders	0	0	1 (1.7)	0
Scrotal pain	0	0	1 (1.7)	0
Respiratory, thoracic, and mediastinal disorders	2 (3.0)	0	0	0
Bronchospasm	1 (1.5)	0	0	0
Hypoxia	1 (1.5)	0	0	0
Skin and subcutaneous tissue disorders	1 (1.5)	0	0	0
Other: Skin lesion	1 (1.5)	0	0	0
Vascular disorders	1 (1.5)	0	3 (5.1)	0
Hypotension	0	0	1 (1.7)	0
Thromboembolic event	1 (1.5)	0	2 (3.4)	0
Any AE	64 (95.5)	0	54 (91.5)	1 (1.7)
Intensification (HD‐MTX)				
Any grade 3–4 event	38 (73.1%)		23 (54.8%)	
	P = 0.083			
Grade 3–4 infection	9 (17.3%)		0 (0%)	
	P = 0.004			
Grade 3–4 nervous system disorders	1 (1.9%)		1 (2.4%)	
	P > 0.99			
On‐therapy mortality	0		0	
Patients who stopped trial treatment due to toxicity	1		0	
Transplant (conditioning and up to Day +30)				
Any grade 3–4 event	38 (100.0%)		20 (90.9%)	
	P = 0.13			
Grade 3–4 infection	13 (34.2%)		6 (27.3%)	
	P = 0.77			
Grade 3–4 nervous system disorders	0		0	
Death during conditioning or within 30 days of SCT 3	2		0	
Toxic epidermal necrolysis	1		0	
Adult respiratory distress syndrome	1		0	
Consolidation blocks 1–4				
Any grade 3–4 event	13 (100%)		19 (100%)	
Grade 3–4 infection	4 (30.8%)		8 (42.1%)	
	P = 0.71			
On‐therapy mortality	0		0	
Grade 3–4 nervous system disorders	1 (7.7%)		0	
	P = 0.41			
Patients who stopped trial treatment due to toxicity	0		0	
Maintenance				
Patients who stopped trial treatment due to toxicity	0		4	

Time to relapse was analyzable for the 142 patients who achieved CR, with 44 events occurring at a median of 11.3 months (IQR: 6.9–20.0). The 3‐year CIR was 28.9% (95% CI: 19.5–41.5) in the SOC + N arm versus 31.0% (95% CI: 21.8–42.9) in the SOC arm: HR 0.81 (95% CI: 0.45–1.48) and P = 0.50 (Figure 2). Site of relapse was available for 43/44 events; 29 involved the bone marrow (BM; 11 SOC + N, 18 SOC) and 9 were isolated to the CNS (7 SOC + N and 2 SOC), with the remaining events occurring in an extramedullary site alone (1 SOC + N and 4 SOC) (Table 2). There was no significant difference in either BM or CNS relapse rates between the 2 arms; the 3‐year cumulative incidence of BM relapse was 16.8% (95% CI: 9.7–28.3) in SOC+N versus 23.3% (95% CI: 15.2–34.8) in SOC, HR 0.62 (95% CI: 0.29–1.32), P = 0.21), and the 3‐year cumulative incidence of CNS relapse risk was 10.8% (95% CI: 5.5–20.4) in SOC + N vs. 4.1% (95% CI: 3.1–12.2) in SOC, HR 2.66 (95% CI: 0.69−10.26), P = 0.14. None of the patients with a CNS relapse had confirmed CNS disease at diagnosis. No diagnostic risk factors for CNS relapse were identified, including WCC ≥100 × 10^9^ L, presence of extramedullary disease (or site of extramedullary disease), or CGN risk group.

Although more SCTs were performed in the SOC arm (54 vs. 34), we did not observe a systematic bias for this; more patients were taken off trial in phase 1 (before Nelarabine was administered) in SOC due to poor response (7 vs. 2), of whom 6 received alloSCT. Among those with disease, more received alloSCT within SOC, 86.4%, than SOC + N, 65.8% (P = 0.027), but this 7‐patient difference was mostly explained by 2 additional patients without suitable donors in the SOC+N arm and 4 SOC + N patients who were planned for SCT but relapsed before it could be administered. Within the standard‐risk group, there was no indication that additional allo SCTs were allocated to SOC patients (details in Supporting Information S1: Table S3). When alloSCT was included as a time‐dependent covariate, conclusions remained the same, with an EFS HR: 0.86 (0.46–1.61), P = 0.64.

Relapses tended to occur early, with 40 of the 44 relapses (90.9%) reported within 2 years of diagnosis. Only four events in total occurred after 3 years from diagnosis for the whole cohort: two deaths in remission and two late relapses. The outcomes after relapse were extremely poor, with 41 of the 44 patients who relapsed having died at the time of reporting. The median survival from relapse was 5.7 months (IQR: 3.0–13.7), with no difference by randomized arm HR = 1.06 (0.57–1.98), P = 0.86 (Figure 2). Treatments used in relapse were wide‐ranging, but included reinduction chemotherapy in 27 patients with or without nelarabine if not previously administered (*N* = 7). A small number of patients also received experimental treatments such as daratumumab (*N* = 1), selumetinib (*N* = 1), venetoclax (*N* = 2), and navitoclax (*N* = 1), as well as stem cell transplant (*N* = 8) and donor lymphocyte infusions (*N* = 2). Two patients also received radiotherapy as part of their salvage regimens. Of the eight patients who received alloSCT after first relapse, three were second allo SCTs. All patients died post‐transplant, with a median survival of 17.5 months from infusion.

Diagnostic DNA samples from 115 patients were analyzed for ABD status. Fifty‐three had ABD of the TCR locus, 52 had a homozygous deletion (non‐ABD), and 10 patients had an indeterminate ABD status. The characteristics of patients by ABD status are shown in Supporting Information S1: Table S4. The median age of patients with non‐ABD status was 34 years versus 42 years in those with ABD, P = 0.25, with fewer >40 (32.7% vs. 52.8%, P = 0.049). Consistent with the more immature level of differentiation arrest,^15^ patients with ABD were less likely to have a target for MRD analysis (18/47, 38.3% with no identifiable MRD target) compared to the non‐ABD group (1 of 52, 1.9%, P < 0.001). The MRD negativity rates at the end of induction 2 for those with a trackable MRD marker were 12/29 (41.4%) in the ABD group versus 35/51 (68.6%) in the non‐ABD group (P = 0.020). Among the 105 patients who were analyzable for ABD status, there were 30 relapses: 10 in the non‐ABD group and 20 in the ABD group HR: 1.97 (0.94–4.11), P = 0.066. The 3‐year relapse rates were 19.5% (95% CI: 11.0–33.2) in the non‐ABD group and 37.3% (95% CI: 25.6–52.0) in the ABD group. We did not demonstrate a benefit of nelarabine according to ABD status in our patient cohort (Supporting Information S1: Figure S2).

DNA from 133 patient diagnostic samples was analyzed for mutations in *NOTCH1, FBXW7, NRAS, KRAS,* and *PTEN* genes (Supporting Information S1: Table S5). There were no significant differences in CR rates or survival outcomes in patients with wild‐type (WT) *NOTCH1* or *FBXW7* compared to patients with single or double mutations in these genes (Supporting Information S1: Table S5). Twelve patients had detectable mutations in *NRAS* at presentation. This small cohort had inferior rates of remission induction (P = 0.022) and inferior EFS compared to patients with WT *NRAS* (P = 0.031).

## CONCLUSIONS

We demonstrated that although one cycle (three doses) of nelarabine can be safely added to induction chemotherapy with no increase in toxicity when treating adults with T‐ALL, we did not observe a relapse reduction (BM or CNS) or survival benefit for patients randomized to receive nelarabine. The Children's Oncology Group have shown a disease‐free survival benefit when nelarabine (six 5‐day courses of 400–650 mg/m^2^/d) was added to augmented BFM therapy in the treatment of children and young adults with newly diagnosed T‐ALL without increased toxicity.^32^ The nelarabine dosing schedule was much lower in UKALL14 and may have been inadequate to result in improved outcomes.

We were cautious about both dosing and administration in our trial design, due to concerns regarding toxicity, excluding any patients with pre‐existing neurotoxicity grade 2 or higher from receiving nelarabine. Ultimately, only 47 of 68 patients randomized to SOC + N received any drug. However, analysis by treatment received rather than intention to treat also failed to show a benefit in EFS in patients receiving nelarabine.

Although there was no difference in EFS between the two treatment arms, the 3‐year EFS for the T‐ALL SOC arm in UKALL14 was superior to that reported for patients treated in the B‐cell arm (58.2% vs. 43.7%).^23^ The 5‐year OS of 59.3% was also an improvement on the 48% reported for the T‐ALL cohort in our previous trial UKALL 12,^1^ despite the considerably older patient population (median age 38 vs. 29 years). Changes made to the SOC chemotherapy regimen to be more consistent with “pediatric” approaches, including switching from native asparaginase to pegylated asparaginase, and modifications to phase II induction allowing patients to progress more rapidly to high‐dose methotrexate intensification benefited the entire UKALL14 T‐ALL cohort.

ETP ALL, as defined by immunophenotyping, was recognized by the WHO in 2016 as a distinct entity within T‐ALL^33^ and was originally reported as being associated with inferior survival outcomes compared to patients with non‐ETP T‐ALL.^12^ ETP ALL is more likely to be resistant to induction therapy and to have a poor early MRD response. Recent studies indicate comparable outcomes for pediatric patients with ETP ALL compared to non‐ETP T‐ALL when treated on risk‐adapted protocols.^34^, ^35^, ^36^ An “ETP”‐like leukemia, defined by gene expression profiling with superior predictive value for treatment failure, has been described more recently,^16^ although there is discordance in patient identification, with only 71% of ETP cases defined by phenotype as having an ETP‐like gene expression profile.^37^ Diagnostic immunophenotyping results were not collected centrally in UKALL14, and so data on the incidence of patients with an ETP phenotype are not available for this cohort. However, patients were analyzed for ABD status as a “surrogate” for ETP gene expression profiling,^15^ acknowledging that ABD, immunophenotypic ETP‐ALL, and gene‐expression‐defined ETP‐like T‐ALL are overlapping but nonequivalent entities. Patients with ABD were older than patients with non‐ABD status and consequently more likely to be in the UKALL14 high‐risk group. Patients with ABD were less likely to be MRD‐negative at the end of induction 2, although this was primarily driven by the inability to identify an MRD marker in this group. ABD was not identified as an independent risk factor for poor outcomes in our adult patient cohort. Early T‐cell progenitors express high levels of *SAMHD1*, a gene recently shown to mediate resistance to nelarabine, suggesting a potential benefit for nelarabine in patients with non‐ABD status.^38^, ^39^ We did not demonstrate a benefit of nelarabine according to ABD status in our patient cohort, but the study was not powered for this purpose.

The previously published 5‐gene classifier has been applied across adult and pediatric cohorts,^18^ although it has not retained its predictive value in all studies.^21^ We were not able to show a predictive value of the 5‐gene classifier in our UKALL14 patient cohort, although we did demonstrate inferior outcomes for the small group of patients with *NRAS* mutations at diagnosis, in line with data from other groups.^22^ More recently, Simonin et al. proposed a novel NGS‐based classifier for T‐ALL, which improved the prognostic value of the original gene classifier with additional incorporation of presenting WCC and postinduction MRD assessment,^40^ and it is likely that this type of prognostic classifier will be used in future trial design to further risk‐adapt therapy for newly diagnosed patients. We highlight the European Working Group for Adult ALL Prognostic Index (EWALL‐PI) risk score, incorporating WCC, genetics, and end of induction MRD^41^ derived from this data set and validated in Italian and Spanish trial data, as a valuable risk classifier.

Relapses in adult patients with T‐ALL treated on UKALL14 mostly occurred early, in line with data from pediatric and adolescent trials,^42^ with only 2 relapses occurring more than 3 years from trial entry. However, outcomes after relapse in this study were dismal, despite some patients accessing novel agents such as daratumumab^43^ and venetoclax/navitoclax.^44^ These data highlight an urgent need for clinical trials in this area and we look forward to outcomes of current CAR‐T trials in patients with relapsed/refractory T‐ALL.

## AUTHOR CONTRIBUTIONS

**Clare J. Rowntree**: Conceptualization; writing—original draft; investigation; writing—review and editing. **Amy A. Kirkwood**: Conceptualization; investigation; formal analysis; writing—original draft; data curation. **Nadine Farah**: Writing—review and editing; data curation; methodology. **Emma Lawrie**: Project administration. **David I. Marks**: Writing—review and editing; investigation. **Andrew K. McMillan**: Writing—review and editing; investigation. **Tobias F. Menne**: Investigation; writing—review and editing. **Anthony V. Moorman**: Investigation; writing—review and editing. **Bela Patel**: Investigation; writing—review and editing. **Pip Patrick**: Data curation; project administration. **Paul Smith**: Project administration. **Krisztina Z. Alapi**: Methodology; data curation; project administration. **Lingyi Wang**: Formal analysis; methodology. **Marc R. Mansour**: Investigation; data curation; writing—review and editing. **Adele K. Fielding**: Conceptualization; investigation; funding acquisition; writing—original draft; resources; writing—review and editing.

## CONFLICT OF INTEREST STATEMENT

AKF is an Editor of *HemaSphere*. The other authors declare no conflicts of interest.

## FUNDING

Cancer Research UK CRUK/A13920 to AKF; CRUK/A21019 to AKF and AVM; Rosetrees Trust PGS19‐2/10152 to NF, AKF, and MM; Great Ormond Street Children's Charity Professorship to MM.

## Supporting information


**Table S1: List of trial centers and principal investigators. Table S2: Main outcomes for all eligible randomized patients. Table S3: Details of post‐induction therapy by risk group. Table S4: Baseline characteristics of patients with ABD and non‐ABD status. Supplementary table S5: Efficacy by genetic subtypes. Supplementary figure 1: trial treatment details. Supplementary figure S2: Nelarabine and EFS within ABD/non‐ABD (interaction p = 0.77) and 5‐gene classifier groups (interaction p = 0.31)**.

## Data Availability

The data that support the findings of this study are available on request from the corresponding author. The data are not publicly available due to privacy or ethical restrictions.
