# Supplementary material for: One cycle of nelarabine given after induction is well tolerated but does not improve outcome in adults with T‐cell precursor acute lymphoblastic leukemia: Outcome data from the UKALL14 randomized‐controlled trial
Source: Hemasphere. 2026 Oct 1;10(10):e70480. doi: 10.1002/hem3.70480 (PMC13628557; doi:10.1002/hem3.70480)
Supplement: Supplementary file 1 [file HEM3-10-e70480-s001.docx]

**Supplementary information**

*One cycle of nelarabine given after induction is safe but does not improve outcome in adults with T cell precursor acute lymphoblastic leukemia - outcome data from the UKALL14 randomized controlled trial.*

**Table S1: List of trial centres and principal investigators**

| Centre | Number of patients | Principal investigator(s) |
| --- | --- | --- |
| Aberdeen Royal Infirmary | 2 | Dominic Culligan |
| Addenbrookes Hospital | 4 | Ram Malladi, Kiran Tawana and Jenny Craig |
| Arrowe Park Hospital | 1 | Ranjit Dasgupta |
| Beatson West of Scotland Cancer Centre | 3 | Mhairi Copland |
| Belfast City Hospital | 5 | Damian Finnegan and Mary-Frances McMullin |
| Blackpool Victoria Hospital | 4 | Paul Cahalin |
| Bradford Royal Infirmary | 1 | Anshu Garg, Lisa Newton, Samuel Ackroyd and Nandini Sadasivam |
| Bristol Haematology and Oncology Centre | 3 | David Marks |
| Castle Hill Hospital | 6 | Simone Green, Sahra Ali and Andrew Fletcher |
| Cheltenham General Hospital | 1 | Adam Rye |
| Christie Hospital | 5 | Anna Castleton and Samar Kulkarni |
| Freeman Hospital | 4 | Tobias Menne and Matthew Collin |
| Hammersmith Hospital | 4 | Jiri Pavlu |
| Hillingdon Hospital | 1 | Richard Kaczmarski |
| Ipswich Hospital | 1 | Debo Ademokun |
| James Cook University Hospital | 1 | Dianne Plews |
| John Radcliffe Hospital | 3 | Andy Peniket |
| Kings College Hospital | 5 | Deborah Yallop and Robert Marcus |
| Leicester Royal Infirmary | 4 | Murray Martin and Ann Hunter |
| Manchester Royal Infirmary | 3 | Eleni Tholouli and John Ahman Liu Yin |
| Monklands Hospital | 1 | Lindsay Mitchell |
| Musgrove Park Hospital | 2 | Simon Bolam |
| New Cross Hospital | 2 | Supratik Basu and Sunil Handa |
| Northwick Park Hospital | 2 | Vaitsa Katsomitrou and Nicki Panoskaltsis |
| Nottingham City Hospital | 7 | Andrew McMillan |
| Pinderfields Hospital | 2 | Paul Moreton |
| Poole General Hospital | 4 | Darshayani Furby and Fergus Jack |
| Queen Alexandra Hospital | 4 | Mary Ganczakowski |
| Queen Elizabeth Hospital Birmingham | 6 | Sridhar Chaganti and Mark Cook |
| Royal Bournemouth Hospital | 1 | Joseph Chacko and Rachel Hall |
| Royal Cornwall Hospital | 1 | Bryson Pottinger |
| Royal Devon and Exeter Hospital | 1 | David Veale and Jackie Ruell |
| Royal Free Hospital/UCLH | 16 | Adele Fielding and Asim Khwaja |
| Royal Hallamshire Hospital | 6 | Nicholas Morley |
| Royal Liverpool University Hospital | 2 | Arpad Toth, Professor Richard Clark, Usira Vithanarachchi, Rahuman Salim and Amit Patel |
| Royal Marsden Hospital London | 11 | David Taussig and Michael Potter |
| Royal United Hospital Bath | 1 | Christopher Knecthli |
| Russells Hall Hospital | 1 | Craig Taylor |
| Sandwell General Hospital | 1 | Yasmin Hasan |
| Singleton Hospital, Swansea | 1 | Unmesh Mohite |
| Southampton General Hospital | 2 | Deborah Richardson and Kim Orchard |
| St Bartholomew’s Hospital | 7 | Matthew Smith and Heather Oakervee |
| St George's Hospital London, London | 4 | Matthisa Klammer |
| St James University Hospital | 4 | Richard Kelly and David Bowen |
| University Hospital Coventry | 1 | Beth Harrison |
| University Hospital Wales | 1 | Clare Rowntree |
| University Hospital of North Stafford.. | 1 | Paul Ferguson and Srinivas Pillai |
| Western General Hospital Edinburgh | 1 | Huw Roddie |
| Whiston Hospital | 1 | Toby Nicholson |
| Ysbyty Gwynedd | 1 | Earnest Heartin, Sally Evans and Jim Seale |

**Table S2: Main outcomes for all eligible randomised patients**

|  |  | **SOC**  **N=81** | **SOC + N**  **N=75** |
| --- | --- | --- | --- |
| **Event-Free survival** | |  |  |
|  | Number of events/patients | 35/81 | 30/75 |
|  | HR (95%CI)  p-value | 0.91 (0.56 – 1.48)  p=0.69 | |
|  | 3-year EFS rate (95%CI) | 58.8% (47.3 – 68.7) | 62.2% (50.1 – 72.2) |
|  |  |  |  |
|  | **Adjusted for baseline risk factors^1^**  HR (95%CI)  p-value | 1.04 (0.58 – 1.87)  p=0.89 | |
|  |  |  |  |
| **Overall survival** | |  |  |
|  | Number of events/patients | 33/81 | 28/75 |
|  | HR (95%CI)  p-value | 0.89 (0.54 – 1.48)  p=0.65 | |
|  | 3-year EFS rate (95%CI) | 63.8% (52.3 – 73.3) | 69.0% (52.3 – 73.3) |
| **Relapse** | |  |  |
|  | Number of events/patients | 25/80 | 20/73 |
|  | HR (95%CI)  p-value | 0.85 (0.47 – 1.53)  p=0.59 | |
|  | 3-year relapse rate (95%CI) | 29.1% (20.4 – 40.4) | 27.8% (18.9 – 39.8) |
|  |  |  |  |

*^1^Age, sex, WCC, genetic risk group, extramedullary involvement and BMI*

**Table S3: Details of post-induction therapy by risk group**

|  |  | | **SOC** | **SOC+N** |
| --- | --- | --- | --- | --- |
|  |  | | **N=75** | **N=68** |
| **Patients off trial protocol before the end of phase 2** | | | **8** | **8** |
| **Died** | | | **0** | **2** |
| **Relapse** | | | **1** | **1** |
| **Off trial for lack of CR induction (all phase 1)** | | | **7** | **2** |
|  | *Off protocol SCT* | | *6* | *1* |
|  | *Chemotherapy only* | | *1* | *1* |
| **Toxicity** | | | **0** | **1** |
|  | *Died before post induction treatment* | | *0* | *1* |
| **Withdrew consent** | | | **0** | **1** |
|  | *Off protocol SCT* | | *0* | *1* |
| **Subdural hematoma** | | | **0** | **1** |
|  | *Chemotherapy only* | | *0* | *1* |
| **Patients on protocol at the end of phase 2** | | |  |  |
| **High risk** | | | **44** | **37** |
| **SCT (including off protocol SCTs)** | | | **38** | **25** |
| **Chemotherapy alone (including off protocol)** | | | **6** | **12** |
|  | *No donor* | | *4* | *6* |
|  | *Not fit enough* | | *1* | *0* |
|  | *Patient refusal* | | *1* | *1* |
|  | *Not suitable (NOS)* | | *0* | *1* |
|  | *Headed for SCT but relapsed* | | *0* | *4* |
| **Standard Risk** | | | **23** | **22** |
| **SCT** | | | **10** | **7** |
|  | | *To protocol i.e. ≤40, sibling SCT* | *9* | *4* |
|  | | *Clinical decision* | *1* | *1* |
|  | | *Clinical decision based on CALM-AF10 and TP53 deletion/mutation* | *0* | *1* |
|  | | *Unable to tolerate further chemotherapy* | *0* | *1* |
| **Chemotherapy alone (including off protocol)** | | | **13** | **15** |
| **Unknown** | | | **0** | **1** |

**Table S4: Baseline characteristics of patients with ABD and Non-ABD status**

|  |  | **Non-ABD** | **ABD** | **p-value** |
| --- | --- | --- | --- | --- |
|  |  | **N=52** | **N=53** |  |
|  |  |  |  |  |
| **Treatment arm, N(%)** | |  |  |  |
|  | SOC | 25 (48.1) | 25 (47.2) | - |
|  | SOC + N | 27 (51.9) | 28 (52.8) |  |
|  |  |  |  |  |
| **Age (years), median (IQR)**  ***Range*** | | 34 (29 - 42.5)  *25 - 60* | 42 (28 - 51)  *25 - 64* | 0.25 |
|  | |  |  |  |
| **Age group, N (%)** | |  |  |  |
|  | 40 or under at randomisation | 35 (67.3) | 25 (47.2) | 0.049 |
|  | Over 40 at randomisation | 17 (32.7) | 28 (52.8) |  |
| **Sex, N (%)** | |  |  |  |
|  | Male | 40 (76.9) | 38 (71.7) | 0.66 |
|  | Female | 12 (23.1) | 15 (28.3) |  |
| **ECOG, N (%)** | |  |  |  |
|  | 0 | 32 (61.5) | 35 (66.0) | 0.93 |
|  | 1 | 19 (36.5) | 13 (24.5) |  |
|  | 2 | 0 | 5 (9.4) |  |
|  | 3 | 1 (1.9) | 0 |  |
| **Baseline WBC group, N (%)** | |  |  |  |
|  | <30 | 16 (30.8) | 31 (58.5) | 0.13 |
|  | 30-100 | 23 (44.2) | 7 (13.2) |  |
|  | >=100 | 13 (25.0) | 15 (28.3) |  |
|  |  |  |  |  |
|  | Median (IQR)  *Range* | 54.1(16.98 - 103.75) *0.36 - 338.8* | 16.5(7.5 - 117.9) *0.6 - 427.05* | 0.024 |
|  |  |  |  |  |
| **Confirmed CNS disease, N(%)** | | 1 (1.9) | 2 (3.8) | >0.99 |
|  |  |  |  |  |
| **Other extra medullary involvement, N(%)** | | 44 (84.6) | 35 (66.0) | 0.041 |
|  |  |  |  |  |
| **t(4;11)(q21;q23), N (%)** | |  |  |  |
|  | Absent | 48 (100.0) | 43 (100.0) | - |
|  | Missing | 4 | 10 |  |
| **t(11q23), N (%)** | |  |  |  |
|  | Absent | 47 (100.0) | 40 (100.0) | - |
|  | Missing | 5 | 13 |  |
| **HOTR or Near Haploidy, N (%)** | |  |  |  |
|  | Absent | 41 (100.0) | 37 (97.4) | 0.48 |
|  | Present | 0 | 1 (2.6) |  |
|  | Missing | 11 | 15 |  |
| **Complex karyotype, N (%)** | |  |  |  |
|  | Absent | 35 (85.4) | 34 (87.2) | >0.99 |
|  | Present | 6 (14.6) | 5 (12.8) |  |
|  | Missing | 11 | 14 |  |
| **Any UKALL 14 cytogenetic risk factor, N (%)** | |  |  |  |
|  | Absent | 35 (85.4) | 32 (84.2) | >0.99 |
|  | Present | 6 (14.6) | 6 (15.8) |  |
|  | Missing | 11 | 15 |  |
| **Cytogenetic group, N (%)** | |  |  |  |
|  | Complex karyotype | 6 (12.5) | 5 (12.5) | 0.57 |
|  | High hyperdiploid | 0 | 1 (2.5) |  |
|  | Low hypodiploid/Near-triploid | 0 | 1 (2.5) |  |
|  | Other | 42 (87.5) | 33 (82.5) |  |
|  | Missing | 4 | 13 |  |
| **Cytogenetic risk group, N (%)** | |  |  |  |
|  | Genetic good risk | 0 | 1 (2.5) | >0.99 |
|  | Standard risk | 42 (87.5) | 33 (82.5) |  |
|  | High risk | 6 (12.5) | 6 (15.0) |  |
|  | Missing | 4 | 13 |  |
| **Baseline UKALL 14 risk, N (%)** | |  |  |  |
|  | Standard risk | 20 (38.5) | 12 (22.6) | 0.063 |
|  | High risk* | 27 (51.9) | 39 (73.6) |  |
|  | Assumed Standard - missing cytogenetic data | 5 (9.6) | 2 (3.8) |  |
| **Baseline UKALL 14 risk (no age), N (%)** | |  |  |  |
|  | Standard risk | 26 (50.0) | 24 (45.3) | 0.90 |
|  | High risk* | 17 (32.7) | 19 (35.8) |  |
|  | Assumed Standard - missing cytogenetic data | 9 (17.3) | 10 (18.9) |  |

**Supplementary table S5: Efficacy by genetic subtypes**

| **Genetic group** | | **Event Free Survival** | | | **Time to relapse** | | |
| --- | --- | --- | --- | --- | --- | --- | --- |
|  |  | **Events/N** | **HR (95%CI)** | **p-value** | **Events/N** | **HR (95%CI)** | **p-value** |
|  |  |  |  |  |  |  |  |
| **Notch** | |  |  |  |  |  |  |
|  | WT | 23/57 | 1.00 | 0.93* | 18/57 | 1.00 | 0.99* |
|  | Single mutation | 20/55 | 0.87 (0.48 – 1.58) |  | 14/53 | 0.80 (0.40 – 1.60) |  |
|  | Double mutation | 9/21 | 1.13 (0.52 – 2.45) |  | 7/21 | 1.12 (0.47 – 2.69) |  |
|  |  |  |  |  |  |  |  |
| **FBXW7** | |  |  |  |  |  |  |
|  | WT | 47/112 | 1.00 | 0.18* | 34/110 | 1.00 | 0.59 |
|  | Single mutation | 4/16 | 0.60 (0.22 – 1.66) |  | 4/16 | 0.91 (0.32 – 2.57) |  |
|  | Double mutation | 1/5 | 0.39 (0.05 – 2.82) |  | 1/5 | 0.57 (0.08 – 4.19) |  |
|  |  |  |  |  |  |  |  |
|  | WT | 47/112 | 1.00 | 0.18 | 34/110 | 1.00 | 0.67 |
|  | mutated | 5/21 | 0.54 (0.21 – 1.36) |  | 5/21 | 0.82 (0.32– 2.09) |  |
|  |  |  |  |  |  |  |  |
| **Notch/FBXW7** | |  |  |  |  |  |  |
|  | Both WT | 21/50 | 1.00 | 0.94 | 16/50 | 1.00 | 0.92 |
|  | Single notch, FBXW7 WT | 17/43 | 0.90 (0.47 – 1.70) |  | 11/41 | 0.78 (0.36 – 1.69) |  |
|  | FBXW7, notch WT | 2/7 | 0.66 (0.16 – 2.83) |  | 2/7 | 0.98 (0.23 – 4.28) |  |
|  | Double mutant | 12/33 | 0.89 (0.44 – 1.81) |  | 10/33 | 1.01 (0.46 – 2.24) |  |
| **NRAS** | |  |  |  |  |  |  |
|  | WT | 44/121 | 1.00 | 0.025 | 34/120 | 1.00 | 0.16 |
|  | Mutant | 8/12 | 2.32 (1.09 – 4.93) |  | 5/11 | 1.93 (0.75 – 4.94) |  |
| **KRAS** | |  |  |  |  |  |  |
|  | WT | 51/130 | 1.00 | 0.79 | 38/128 | 1.00 | 0.95 |
|  | Mutant | 1/3 | 0.76 (0.10 – 5.50) |  | 1/3 | 1.06 (0.15– 7.74) |  |
|  |  |  |  |  |  |  |  |
| **NRAS/KRAS** | |  |  |  |  |  |  |
|  | Both WT | 43/118 | 1.00 | 0.080 | 33/117 | 1.00 | 0.38 |
|  | NRAS only | 8/12 | 2.31 (1.08 – 4.92) |  | 5/11 | 1.93 (0.75 – 4.96) |  |
|  | KRAS only | 1/3 | 0.83 (0.11 – 6.06) |  | 1/3 | 1.13 (0.16 – 8.30) |  |
|  |  |  |  |  |  |  |  |
|  | Both WT | 43/118 | 1.00 | 0.069 | 33/117 | 1.00 | 0.21 |
|  | Any RAS | 9/15 | 1.93 (0.94 – 3.96) |  | 6/14 | 1.73 (0.72 – 4.14) |  |
|  |  |  |  |  |  |  |  |
| **PTEN point** | |  |  |  |  |  |  |
|  | WT | 49/127 | 1.00 | 0.34 | 39/125 | 1.00 | 0.13 |
|  | Mutant | 3/6 | 1.74 (0.54 – 5.59) |  | 0/6 | - |  |
|  |  |  |  |  |  |  |  |
| **PTEN indel** | |  |  |  |  |  |  |
|  | WT | 48/126 | 1.00 | 0.18 | 37/124 | 1.00 | >0.99 |
|  | Mutant | 4/7 | 1.97 (0.71 – 5.48) |  | 2/7 | 1.00 (0.24 – 4.14) |  |
|  |  |  |  |  |  |  |  |
| **PTEN group** | |  |  |  |  |  |  |
|  | WT | 47/123 | 1.00 | 0.47 | 37/121 | 1.00 | 0.34 |
|  | PTEN point mutation | 1/3 | 1.01 (0.14 – 7.35) |  | 0/3 | - |  |
|  | PTEN indel mutation | 2/4 | 1.52 (0.37 – 6.25) |  | 2/4 | - |  |
|  | PTEN point and indel mutation | 2/3 | 2.82 (0.68 – 11.66) |  | 0/3 | - |  |
|  |  |  |  |  |  |  |  |
|  | WT | 47/123 | 1.00 | 0.28 | 37/121 | 1.00 | 0.53 |
|  | Any mutation | 5/10 | 1.66 (0.66 –4.17) |  | 2/10 | 0.63 (0.15 – 2.63) |  |
|  |  |  |  |  |  |  |  |
| **5 gene classifier** | |  |  |  |  |  |  |
|  | NOTCH/FBXW7 WT and RAS/PTEN WT | 16/43 | 1.00 | 0.049 | 13/43 | 1.00 | 0.83 |
|  | NOTCH/FBXW7 WT and RAS/PTEN Mutant | 5/7 | 2.92 (1.07 – 8.01) |  | 3/7 | 1.63 (0.46 – 5.71) |  |
|  | NOTCH/FBXW7 Mutant and RAS/PTEN WT | 22/66 | 0.90 (0.47 – 1.71) |  | 18/65 | 0.92 (0.45 – 1.88) |  |
|  | NOTCH/FBXW7 Mutant and RAS/PTEN Mutant | 9/17 | 1.68 (0.74 – 3.80) |  | 5/16 | 1.10 (0.39 –3.08) |  |
|  |  |  |  |  |  |  |  |
|  | NOTCH/FBXW7 Mutant and RAS/PTEN WT | 22/66 | 1.00 | 0.18 | 18/65 | 1.00 | 0.62 |
|  | All other groups | 30/67 | 1.45 (0.84 – 2.51) |  | 21/66 | 1.17 (0.62 – 2.20) |  |
|  |  |  |  |  |  |  |  |
| **ABD** | |  |  |  |  |  |  |
|  | Non-ABD | 25/53 | 1.00 | 0.37 | 11/52 | 1.00 | 0.066 |
|  | ABD | 19/52 | 1.31 (0.72 - 2.38) |  | 20/51 | 1.97 (0.94 – 4.11) |  |
|  |  |  |  |  |  |  |  |

**Test for trend*

| **Induction therapy** | **Pre-randomisation** | Dexamethasone pre-phase 5-7 days | |
| --- | --- | --- | --- |
|  | **Phase 1 induction** | Daunorubicin 30mg/ m^2^  Vincristine 1.4mg/ m^2^  Dexamethasone 10mg/m2 four-day blocks  D1,8,15,22  Pegylated asparaginase 1000iu/m^2^ D4 & 18 (over 40 D18 only)  IT methotrexate D14 | |
|  | **Phase 2 induction** | Cyclophosphamide 1g/ m^2^ D1,15  Cytarabine 75mg/ m^2^ 4 days D2,9,16,23  6-mercaptopurine 60mg/ m^2^ daily  IT methotrexate D1,8,15,22 | |
|  | **Nelarabine block** | Nelarabine 1.5g/m^2^ D 1, 3 and 5  Patients randomised to SOC + Nelarabine only | |
| **Risk stratification**   - *High risk cytogenetics: BCR-ABL1, KMT2A-AFF1,* Low hypodiploidy/near-triploidy or Complex karyotype (>5 abn.) - Age 41 years + - Presenting WBC > 30x 10^9^/l - Any MRD+ after phase 2 induction | | | |
| **Post-remission therapy** | **Standard risk** | | **High risk** |
|  | **Intensification**  Methotrexate 3g/m2 combined with pegylated asparaginase 1000 units/m^2^ | | **Sibling or 10/10 MUD alloSCT**  **Patients aged 40 and under: myeloablative conditioning**  1320Gy 8 fractions *plus* etoposide 60mg/kg IV D-3 OR cyclophosphamide 60mg/kg IV D-3 and -2  **Intensification optional if alloSCT is ready to proceed post-induction.**  **Patients aged 41+: reduced intensity conditioning**  Fludarabine 30mg/m^2^ D -7 to D -3, melphalan, 140mg/m^2^ D-1 alemtuzumab 30mg IV D- 2 and D -1 (unrelated donor) or D -1 (matched sib donor) **Intensification always given prior to RIC alloSCT** |
|  | **Consolidation** - **four blocks**  **1**. Etoposide 100mg/ m^2^ D1-5  Cytarabine 75mg/ m^2^ 4 days D 1-5  Pegylated asparaginase 1000iu/m^2^ D5  IT methotrexate D1  **2.** Etoposide 100mg/ m^2^ D1-5  Cytarabine 75mg/ m^2^ 4 days D 1-5  IT methotrexate D1  **3.** as per induction 1 plus 14 days of induction 2  **4.** as per consolidation 2 | |  |
|  | **Maintenance**  2 years of daily oral 6mercaptopurine, weekly oral methotrexate with 3-monthly vincristine/steroid pulses plus intrathecal methotrexate | |  |

**Supplementary figure 1: trial treatment details**

**Supplementary figure S2: Nelarabine and EFS within ABD/non-ABD (interaction p = 0.77) and 5-gene classifier groups (interaction p=0.31)**
